# Synergistic Effects of Combined Dynamic Covalent Bonds and Noncovalent Interactions in Highly Advanced Switchable Adhesive Polymers

**DOI:** 10.3390/polym18060738

**Published:** 2026-03-18

**Authors:** Trong Danh Nguyen, Jun Seop Lee

**Affiliations:** Department of Materials Science and Engineering, College of Engineering, Gachon University, 1342 Seonpolymersnam-daero, Sujeong-gu, Seongnam-si 13120, Republic of Korea

**Keywords:** adhesive polymers, dynamic covalent bonds, noncovalent interactions

## Abstract

Polymer adhesive materials have been utilized across a wide range of applications, including adhesion to wood, metals, and biomaterial substrates. To meet increasing performance demands, the development of high-performance adhesive materials continues to be actively pursued by introducing advanced functions and capabilities into polymer networks. By incorporating dynamic covalent bonds into the polymer network, these materials gain self-healing and reprocessing abilities. While these materials exhibit high mechanical robustness and stability under service conditions, the bonding/rebonding reactions of dynamic covalent bonds allow the polymers to detach from target surfaces when needed. Additionally, noncovalent interactions within the network and between the polymer and the target surface significantly contribute to overall adhesive strength. Although dynamic covalent bonds and noncovalent interactions operate through different mechanisms, both contribute significantly to adhesive performance. This review manuscript presents studies on polymer networks containing dynamic covalent bonds and non-covalent interactions. Based on these studies, the respective contributions of each type of bond to the superior adhesive strength of the materials are discussed, and potential target substrates for adhesion, including wood, metal, and biomaterials, are proposed.

## 1. Introduction

Adhesive materials play crucial roles in the advancement of technologies, both in industry and everyday life [1,2]. Despite their long history, ongoing efforts aim to enhance their properties for diverse applications. For instance, new adhesives have been developed for bonding materials in challenging environments, such as space and underwater. These conditions present unique challenges that require adhesives to possess multiple properties to achieve high adhesive strength [3], particularly robustness [4,5]. In addition to high adhesive strength, advanced adhesive systems are increasingly expected to provide durability, environmental tolerance, and, in some cases, controlled detachment to support material repair, replacement, and reuse [6]. Adhesion relies on two primary mechanisms: mechanical interlocking and chemical interactions [7]. Consequently, polymers are promising candidates for these applications because their superior mechanical strength allows adhesives to withstand applied forces and maintain effective bonding between surfaces [8,9].

**Figure 1:**
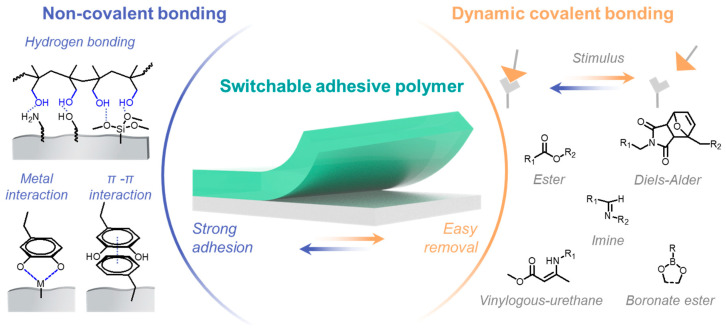
Contributions of noncovalent and dynamic covalent bonds in the switchable adhesive polymers.

Detachment on demand is an intriguing capability that allows adhesive polymers to alter their properties for easier separation from target materials [10], driven largely by the growing need for material reuse [11,12]. However, the stability of traditional polymer networks poses significant challenges. The introduction of dynamic covalent bonds, although a long-standing area of research, continues to be explored for its potential to impart self-healing [13,14] and reprocessing abilities to polymers [15,16,17]. These dynamic covalent bonds offer more than just these two advantages; their reversibility allows polymers to undergo cycles of bonding and rebonding in response to suitable stimuli [18,19]. This reversible network behavior also provides a useful strategy to maintain stability during use while enabling adaptive functions, which is difficult to achieve using permanently crosslinked adhesive networks [20]. In principle, the presence of dynamic covalent bonds can alter the physical properties of adhesive polymers by changing the network morphology and connectivity, depending on the exchange reaction and the applied stimulus [21]. This phenomenon enables adhesive polymers to exist in two distinct states: one characterized by strong adhesive strength and high physical properties, and another with weakened adhesive strength and lower physical properties due to the dissociation of dynamic covalent bonds (Figure 1). Nevertheless, excessive network dynamics may reduce mechanical stability under load, indicating that the exchange behavior of dynamic covalent bonds must be carefully designed for adhesive applications [22].

Non-covalent interactions play crucial roles not only in adhesive strength but also in reinforcing the polymer network and improving resistance to cohesive failure under stress [23]. Research on adhesive materials has shown that these interactions significantly impact the performance of the proposed materials. Depending on their type and distribution, non-covalent interactions can contribute to interfacial adhesion (between the adhesive and substrate), cohesive reinforcement (within the polymer network), and energy dissipation during deformation [24]. Therefore, dynamic covalent bonds and non-covalent interactions can be considered complementary design elements: the former provides reversibility and adaptive network rearrangement, whereas the latter strengthens adhesion and mechanical performance. Their combined use is particularly attractive for advanced adhesive systems that require both high bonding performance and functional responsiveness.

This review introduces studies focused on adhesive polymer materials that incorporate dynamic covalent bonds. Additionally, it discusses the contributions of non-covalent interactions, providing readers with a clear understanding of how dynamic covalent bonds and non-covalent interactions collaborate to achieve exceptional adhesive performance. Accordingly, this review highlights how dynamic covalent bonds and non-covalent interactions jointly influence interfacial adhesion, cohesive strength, mechanical robustness, and controllable debonding behavior. The manuscript summarizes representative adhesive systems and discusses their structure–property relationships and future opportunities for advanced adhesive applications.

## 2. Mechanisms of Adhesion in Switchable Polymer Adhesives

**Figure 2:**
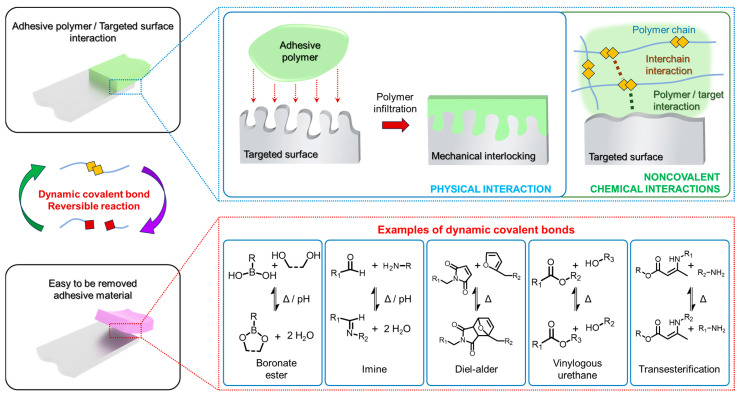
Mechanism of adhesive polymer materials–targeted surfaces interactions and reversible reaction of dynamic covalent bonds.

As written above, there are two primary mechanisms through which adhesive materials achieve strong adhesion: mechanical interlocking and chemical bonding. While these mechanisms can function independently, they both enhance adhesion across a range of materials (Figure 2) [25,26]. Notably, they can also work together to produce exceptional adhesive strength [27]. However, several factors need to be taken into account.

### 2.1. Roles of Dynamic Covalent Bonds

Mechanical interlocking, as the term suggests, relies on the interlocking of adhesive materials with the target surface [28,29]. However, several conditions must be met for effective adhesion. First, the adhesive must penetrate the cracks in the target surface, a topic not covered here. Generally, the rougher the surface, the better the adhesion. Additionally, there is a trade-off regarding the toughness of adhesive material; while high toughness can help maintain stability, sufficient viscosity is also necessary for effective penetration into the surface [30,31]. A straightforward example of this principle can be seen in commercial cyanoacrylate adhesive (super glue). Low-viscosity monomer components are applied to infiltrate cracks, and subsequent polymerization creates a hard layer that bonds multiple surfaces together [32]. However, this bond is permanent, requiring force to break the adhesive, often leaving residue on the surfaces [33]. This characteristic can be disadvantageous when surfaces need to be separated. In contrast, soft adhesive polymers offer adhesion that is easier to undo [34]. A common example is the adhesive used in sticky notes, although its adhesive strength is relatively low.

Introducing dynamic covalent bonds into materials enables a transformation between liquid/gel and solid states [35,36]. The solid state provides strong adhesion, while the liquid/gel state facilitates surface penetration and, if necessary, the release of the target surface. This capability, termed “detach on demand”, is central to switchable adhesive polymers [37]. After detachment, the adhesive can be removed cleanly from the target surface, which is a significant advantage in many applications.

### 2.2. Roles of Weak Noncovalent Interactions

Chemical bonding differs from mechanical interlocking in that it does not rely on the roughness of the target surface [38]. Instead, it is based on the interaction between the adhesive material and the target. Hydrogen-bonding interactions are commonly employed for adhesion, as they do not require any modification of the target surface [39]. Many materials, including metals, wood, and organic tissues, can form hydrogen bonds, resulting in strong connections with adhesive materials and excellent adhesive strength. In cases where adhesion cannot depend on mechanical interlocking—often due to smooth surfaces like glass or metal—these interactions serve as the foundation for effective adhesion.

Moreover, the role of hydrogen bonds extends beyond interfacial interactions; they also affect the physical properties of adhesive polymers [24]. While the physical characteristics of polymers do not directly influence their chemical interactions with target surfaces, they are still crucial for adhesive performance. In numerous cases, adhesion fails not because the adhesive detaches from the target but because the adhesive material itself fails under stress. Here, non-covalent interactions enhance robustness. These interactions include not only hydrogen bonding but also π-π, ionic, and metal-ligand interactions, etc. [40]. Due to the efficiency of hydrogen bonds in engaging adhesion targets, they are frequently used (either intentionally or unintentionally) to adjust the final properties of materials. The following sections on dynamic covalent bonds will provide examples of non-covalent interactions in various studies on adhesive polymers.

## 3. Synergy Between Dynamic Covalent Bonds and Noncovalent Interactions in Polymer Networks

### 3.1. Boronate Esters

**Figure 3:**
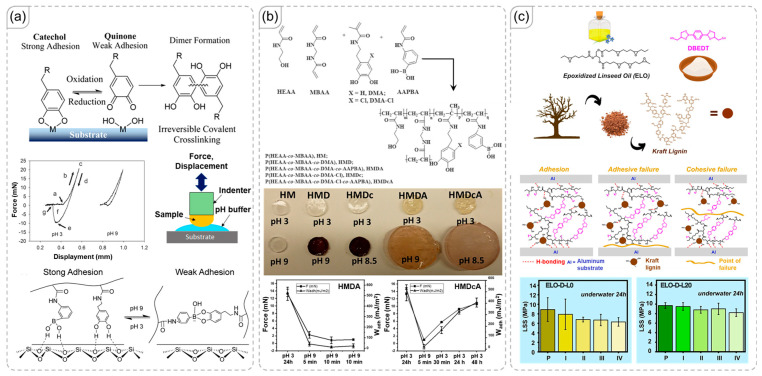
(**a**) Influence of boronate ester exchange on hydrogen-bonding interactions formed by hydroxyl functional groups [45]. Copyright 2016, American Chemical Society. (**b**) Control of adhesive strength through environmental pH, facilitated by the reversible reaction of boronate esters [46]. Copyright 2022, Elsevier. (**c**) Introduction of lignin to create additional hydrogen bonds and enhance material recovery following adhesive failure [47]. Copyright 2024, Elsevier.

One of the most common examples of dual bonding involves the combination of boronate ester formation and hydrogen-bonding interactions [41,42]. Boronic acid functional groups contain hydroxyl groups that can engage in hydrogen bonding with neighboring materials. The dynamic nature of boronate ester formation involves these hydroxyl groups, allowing for control over the adhesive strength of the polymer [43,44]. Specifically, because hydroxyl groups can participate in adhesive interactions with other surfaces, the equilibrium of this reversible reaction directly influences adhesion and other material properties. Narkar et al. clearly discussed this phenomenon in their work (Figure 3a), demonstrating that environmental pH can affect the equilibrium of the boronate ester exchange reaction [45]. They utilized this effect to create a polymer capable of adapting its adhesive strength based on the surrounding pH. Liu et al. advanced this concept further by synthesizing polymer chains from monomers with various functional groups, including boronic acid and hydroxyl groups, as illustrated in Figure 3b [46]. Variations in the chemical network impacted the properties of the polymer and its response to environmental pH. Additionally, the authors performed aromatic ring chlorination of dopamine, which introduced antibacterial properties to the material. Since Cl-catechol can only kill bacteria when unbound from boronic acid, they effectively used boronate ester formation to control the antibacterial activity of the material.

Nardi et al. explored a different approach by using lignin as an intermediate to create hydrogen-bonding crosslinks between the main chains of epoxidized linseed oil (Figure 3c) [47]. When lignin was introduced at an optimal concentration, the adhesive strength was enhanced. Additionally, the presence of lignin improved adhesion to the target surfaces through increased hydrogen-bonding interactions. However, the enhancement was not substantial, likely because epoxidized linseed oil already offers adequate hydrogen-bonding sites for robust interactions with the aluminum surface.

### 3.2. Imines, Oximes, and Hydrazones

Imine, oxime, and hydrazone dynamic covalent bonds are formed from aldehyde functional groups [48]. These groups can be easily derived from carbohydrate polymers, making these polymers promising sources for introducing imine, oxime, and hydrazone bonds [49,50]. Given that most carbohydrate polymers are biocompatible, they are also suitable for biomedical applications [51,52]. Moreover, their networks generate numerous hydrogen-bonding interactions, resulting in high adhesion. However, this strong adhesion comes with the drawback of low physical properties, which can be significantly enhanced through covalent crosslinking [53,54].

**Figure 4:**
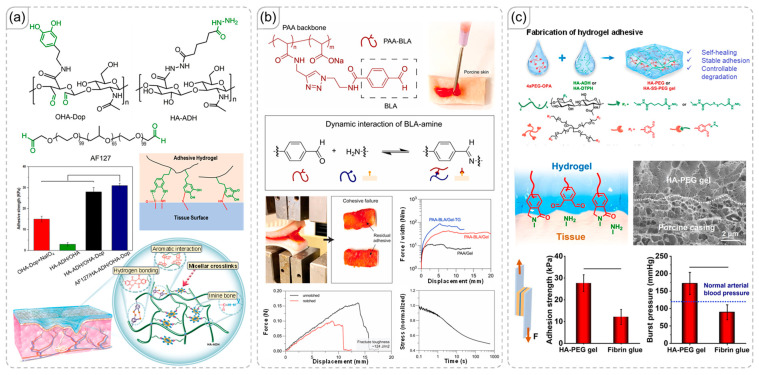
(**a**) Modification of hyaluronic acid to introduce hydrazone dynamic covalent crosslinks [55]. Copyright 2020, American Chemical Society. (**b**) Formation of imine crosslinks and their impact on adhesive properties in tissue adhesive applications [56]. Copyright 2024, Elsevier. (**c**) Adhesive performance of hyaluronic acid-based materials utilizing hydrogen-bonding interactions, dynamic covalent crosslinks, and covalent conjugation with tissue surfaces [57]. Copyright 2023, Science Advances.

In their study, Yang et al. demonstrated that incorporating hydrazone linkages as covalent crosslinking units can create a network structure based on hyaluronic acid, effectively reducing resistance to compressive forces (Figure 4a) [55]. The combination of hydrazone and hydrogen bonding produced exceptional adhesive strength, surpassing both a simply crosslinked network and a network enhanced by hydrogen bonding. Additionally, the authors introduced aldehyde-terminated Pluronic F127 (AF127) for further enhancement. While there was minimal change in adhesive strength, the improvement in physical properties was evidenced by rheological results. Notably, all these benefits were achieved without compromising wound-healing performance.

The publication by Nam et al. exemplifies how imine dynamic covalent bonds can enhance the properties of materials like poly(acrylic acid) in tissue adhesive applications (Figure 4b) [56]. The authors effectively compared networks with and without modifications involving dynamic covalent bonds. In addition to the increased toughness of the polymer network enhanced by dynamic covalent bonds, the tissue adhesive demonstrated significant improvements over the control sample, which relied solely on hydrogen-bonding interactions. Ren et al. explored the innovative concept of substituting some interactions with tissue materials using covalent bonds, as shown in Figure 4c [57]. They employed o-phthalaldehyde, which contains two aldehyde functional groups that can form hydrazone dynamic covalent bonds and stable covalent conjugations with amine groups in tissue materials. Nevertheless, hydrogen-bonding interactions continued to play a significant role in influencing the viscosity of material, enabling it to penetrate complex tissue surfaces and form the strong covalent bonds mentioned earlier.

### 3.3. Diels-Alder Chemistry

**Figure 5:**
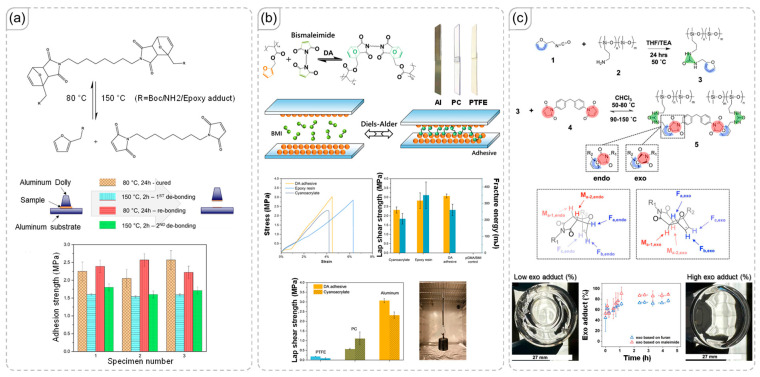
(**a**) Introduction of Diels-Alder (DA) units into an epoxy network and the recovery of adhesion after repeated bonding-debonding cycles [66]. Copyright 2023, Elsevier. (**b**) The effect of DA on the adhesive performance of a polymer [67]. Copyright 2025, American Chemical Society. (**c**) Conversion of endo to exo adducts in the polymer and its resulting influence on material properties [68]. Copyright 2025, American Chemical Society.

Diels-Alder (DA) bonds are dynamic covalent bonds valued for their reversible reactions, which can be easily controlled [58,59]. Both the forward and retro DA reactions occur within different temperature ranges that current technologies can readily achieve [60,61,62]. However, the slow reaction rate poses a significant challenge, limiting network formation [63]. To address this, many researchers incorporate DA units into networks as pre-formed DA adducts [64,65]. Essentially, DA bonds are initially formed in small molecules before being integrated into the polymer network. An illustrative example of this approach is provided by Ramimoghadam et al. (Figure 5a) [66]. After multiple cycles of debonding and rebonding, the material maintained a good level of adhesion strength, despite potential changes in the network during the dissociated state of the DA bonds. Notably, the adhesive performance of the polymer was not significantly enhanced by the DA bonds themselves, but rather depended on non-covalent interactions with the target surface. In contrast, the modified poly(glycidyl methacrylate) main chain studied by Gou et al. demonstrated significantly higher adhesive strength across various surfaces, including aluminum and polymers like poly(tetrafluoroethylene) and polycarbonate (Figure 5b) [67].

In DA systems, bonds formed from maleimide and furan can exist in two configurations: endo and exo. Both configurations can reversibly bond and debond when exposed to appropriate temperatures. However, the exo adduct is significantly more stable than the endo adduct. Specifically, the endo adduct dissociates at a lower temperature than the exo adduct, and this trend is also observed in their rebonding temperatures. Typically, the materials studied contain both endo and exo adducts, leading to a broad temperature range for the reversible reaction. Several efforts have been made to control the ratio of DA configurations, with the work of Moreau et al. serving as a notable example (Figure 5c) [68]. As noted in their manuscript, the conversion of endo to exo can be achieved by maintaining the system at an optimized high temperature of 80 °C. Increasing the exo content also enhances properties such as shear strength and transparency.

### 3.4. Vinylogous/Hindered Urethane Exchange

**Figure 6:**
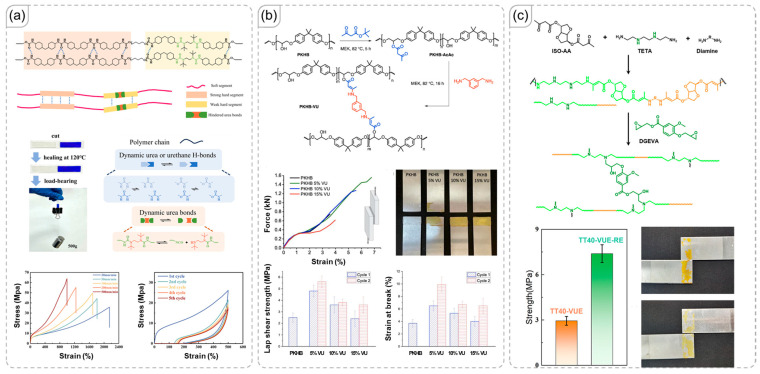
(**a**) Hydrogen interactions in a hindered urethane polymer network that enable a self-healing, ultra-stretchable structure [76]. Copyright 2024, American Chemical Society. (**b**) The influence of vinylogous urethane dynamic crosslinks on the physical properties and adhesive strength of material [77]. Copyright 2023, Elsevier. (**c**) The role of vinylogous urethane in the main chain and the impact of covalent crosslinking on adhesive performance [78]. Copyright 2023, American Chemical Society.

Urethane typically forms irreversible covalent bonds between isocyanate and hydroxyl functional groups [69]. However, specific types of urethane linkages, such as vinylogous urethane (derived from acetoacetate and amine) and hindered urethane, can participate in reversible reactions [70,71,72,73]. The structure of urethane networks also facilitates significant hydrogen bonding [74,75]. Although Hao et al. did not emphasize adhesive properties, their research exemplifies the interaction between hindered urethane chemistry and hydrogen bonding (Figure 6a) [76]. Hindered urethane allows for self-healing, while hydrogen bonding creates hard segments, resulting in impressive stretchability of up to 1693% strain due to the coexistence of soft and hard segments. Additionally, the extensive hydrogen bonding likely contributes significantly to adhesive strength.

Similarly, the study by Soavi et al. illustrated the connection between vinylogous urethane dynamic covalent crosslinks and their effect on the properties of adhesive polymers (Figure 6b) [77]. An optimal level of covalent crosslinking enhances the properties of polymer, including lap shear strength and strain at break. However, increasing the crosslink density beyond a certain point diminishes these properties. Therefore, it is crucial to manage crosslink concentration to achieve optimal performance. When vinylogous urethane units are integrated into the main-chain structure, conventional covalent crosslinking can also be employed to adjust network properties, as demonstrated by Yang et al. (Figure 6c) [78]. A high concentration of vinylogous urethane units in the main chain promotes reversible exchange and related functionalities, such as self-healing or degradation. Nevertheless, the authors employed conventional covalent crosslinking through an epoxide ring-opening reaction to enhance the physical properties. Hydrogen-bonding interactions provided the adhesive material with stability by increasing viscosity during epoxy ring-opening, thereby ensuring excellent adhesive strength.

### 3.5. Transesterification

**Figure 7:**
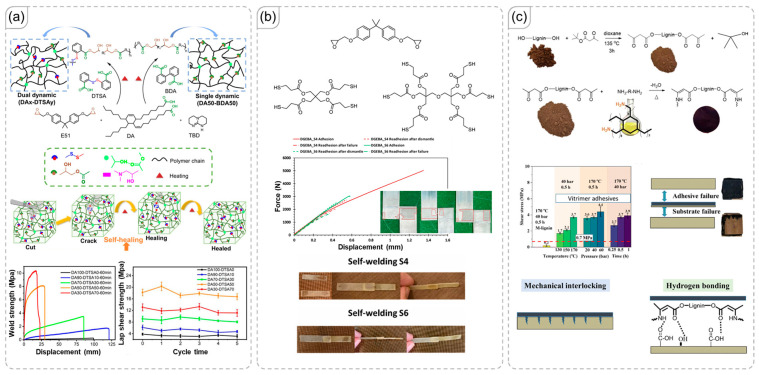
(**a**) Polymer network designed for transesterification in adhesive applications [80]. Copyright 2024, American Chemical Society. (**b**) Comparison of adhesive performance between four-arm and six-arm crosslinkers, each containing ester bonds in their arms [84]. Copyright 2023, Elsevier. (**c**) Lignin-based adhesive material, illustrating the roles of covalent crosslinking and hydrogen bonding in the adhesion mechanisms and wet bonding strength of materials, which surpassed the standard of 0.7 MPa (red dashed line) [85]. Copyright 2023, Royal Society of Chemistry.

Like urethane, ester structures promote the formation of a high concentration of hydrogen-bonding interactions [79]. Huang et al. suggested that hydrogen-bond-augmented physical crosslinks enhanced tensile strength by improving stress dissipation (Figure 7a). Stress relaxation, the ability of a material to reduce internal stress and adapt over time, is a crucial property that warrants investigation. The authors demonstrated that materials with a higher concentration of these interactions exhibited excellent stress-relaxation capabilities and stretchability [80].

Transesterification not only allows for reprocessing, self-healing, and welding but also enables detachable adhesive behavior [81,82,83]. Roig et al. used two crosslinkers to create polymer networks capable of undergoing transesterification: one with four arms (pentaerythritol tetrakis(3-mercaptopropionate)) and another with six arms (dipentaerythritol hexakis(3-mercaptopropionate)), both containing thiol functional groups (Figure 7b) [84]. The six-arm crosslinker, with its greater density of ester bonds, showed superior self-healing ability. However, its physical properties and adhesive strength were inferior to those of the four-arm sample, attributed to the higher chain mobility in the four-arm network, which facilitated more efficient stress relaxation. Furthermore, the four-arm crosslinker likely generated fewer non-covalent interactions, such as hydrogen bonding.

In a different approach, Liu et al. modified lignin to introduce ester groups that enable transesterification, as illustrated in Figure 7c [85]. Lignin, a carbohydrate-rich material, features abundant hydrogen-bonding interactions. The authors clarified the distinct roles of covalent crosslinking and hydrogen bonding: while hydrogen bonding interacts with the adhesion surface, covalent crosslinking stabilizes the interlocking mechanism, significantly enhancing the adhesive strength of material.

## 4. Applications

There are multiple applications in which adhesive materials can be utilized, as the incorporation of dynamic covalent bonds and non-covalent interactions can, in principle, provide effective adhesion on a wide range of surfaces, including wood, metal, glass, plastics, and biomaterials. However, glass and polymeric materials generally possess smooth surfaces, which provide fewer opportunities for mechanical interlocking, an important factor in achieving strong adhesion. As a result, the number of studies in these areas remains limited compared with the other substrate categories discussed in this work.

### 4.1. Wood Adhesives

**Figure 8:**
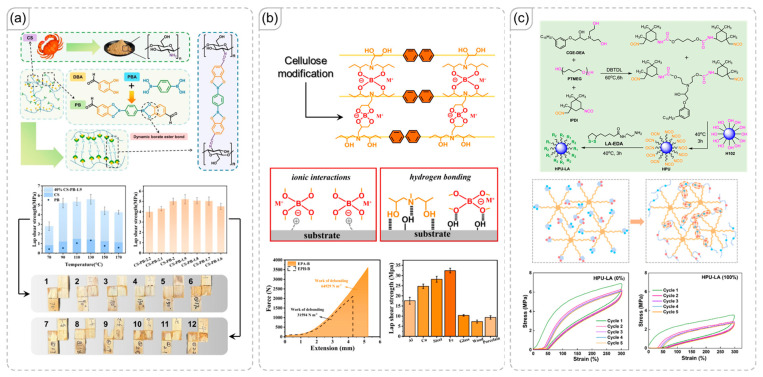
(**a**) Introduction of boronate ester dynamic covalent bonds into chitosan for wood adhesive applications [91]. Copyright 2024, Elsevier. (**b**) Boronate ester modification of cellulose to create a wood adhesive material [92]. Copyright 2025, American Chemical Society. (**c**) Disulfide dynamic bonding enabling rebonding and detach-on-demand behavior, introduced through urethane formation [95]. Copyright 2025, American Chemical Society.

Wood materials, which are primarily composed of carbohydrates, contain a high density of hydroxyl functional groups. These hydroxyls serve as effective bonding sites, particularly in wood adhesive applications [86,87]. Covalent bonds formed between wood hydroxyls and compatible functional groups can achieve significant adhesive strength [88]. When these bonds are dynamic covalent bonds, they allow for detachment on demand within the network [89]. Conversely, hydrogen bonding is crucial for adhesion to other materials such as glass, metal, or ceramics [90]. This concept underpins the research conducted by Yu et al., who modified chitosan to incorporate boronate ester units (Figure 8a) [91]. These boronate esters readily form with the hydroxyl groups on cellulose, presenting a promising method for enhancing adhesion to wood while preserving a considerable amount of hydrogen bonding interactions. Similarly, Liu et al. focused on cellulose to create an adhesive material utilizing dynamic covalent bonds from boronate esters (Figure 8b) [92]. The combination of hydrogen bonding and boronate ester formation results in desirable durability for these adhesive materials.

In addition to boronate esters, urethane linkages can also be formed from hydroxyl groups. However, as noted earlier, only vinylogous and hindered urethane linkages are capable of undergoing reversible reactions, which restricts the potential of urethane in detach-on-demand systems. Nonetheless, urethane chemistry offers straightforward modification opportunities for introducing other dynamic bonds [93,94]. In the study by Zhou et al., urethane linkages were employed to introduce disulfide bonds, which facilitated self-healing and detach-on-demand capabilities (Figure 8c) [95]. A limitation of this approach, however, is that disulfide bonds, unlike the previous examples, do not establish strong interactions with wood surfaces. Instead, the interaction with the wood surface primarily relies on hydrogen bonding from the urethane groups.

### 4.2. Metal Adhesives

**Figure 9:**
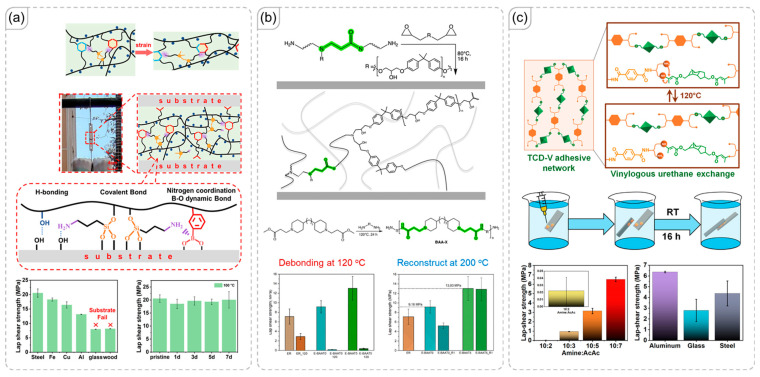
(**a**) Polymer network with boronate ester bonds, which can form stable covalent bonds with steel substrates [102]. Copyright 2023, American Chemical Society. (**b**) Polymer network with transesterification capabilities, offering hydrogen-bonding interactions for adhesion to steel surfaces [103]. Copyright 2025, Royal Society of Chemistry. (**c**) 3D polymer networks created from washed polyethylene terephthalate, facilitating underwater adhesion to metal surfaces [106]. Copyright 2025, Science Advances.

Metal materials often feature slightly rough or smooth surfaces, which makes adhesion heavily reliant on hydrogen-bonding interactions [96,97,98]. Consequently, polar functional groups like hydroxyl or boronic acid are preferred for enhancing metal adhesion [99]. Additionally, certain agents can improve bonding with metal substrates; one notable example is 3-aminopropyltriethoxysilane, which can form covalent bonds with steel [100,101]. This mechanism is central to the research conducted by Zheng et al., who introduced 3-aminopropyltriethoxysilane into a polymer network through covalent bonding (Figure 9a) [102]. Their method resulted in a network that established strong bonds with the steel surface. Moreover, the fabricated network included hydroxyl and boronic ester groups, which acted as sources for boronate ester crosslinking. However, the silane did not produce stable crosslinks, allowing for reprocessing enabled by boronate ester exchange. Although the outcomes were impressive, this design obscured the cooperative effects of dynamic covalent and non-covalent interactions. Therefore, the work of Portone et al. (Figure 9b) was also examined [103]. They utilized β-aminoamide dynamic exchange chemistry based on transesterification, enabling the material to undergo exchange reactions while maintaining excellent adhesive performance [104]. This research achieved remarkable lap shear strength, despite the absence of stable covalent bonds between the polymer and the steel surface.

While transesterification typically requires high temperatures, its reversible-reaction advantage can be challenging to exploit under everyday conditions. Nevertheless, it can be employed in various ways [105]. For instance, Danielson et al. modified washed PET into amphiphilic polyethylene terephthalate terminated with amine groups. These amine-terminated molecules (shown in orange) were then reacted with a series of acetoacetate-terminated molecules (shown in green) to create vinylogous urethane linkages, as illustrated in Figure 9c [106]. This design introduced two types of dynamic covalent bonds within the network. By adjusting the quantity of acetoacetate molecules, the morphology of the 3D polymer network could be fine-tuned, consequently altering its physical properties and hydrogen-bonding interactions. As a result, the adhesive strength varied with changes in network morphology, particularly concerning metals like aluminum and steel, as well as glass. Notably, these materials can also provide adhesion in aqueous environments due to their water-repellent characteristics.

### 4.3. Biomaterials Adhesives

Adhesion materials for biomaterials have specific requirements due to their application environments. The primary requirement is that the polymer must be biocompatible, meaning it should not induce toxic, inflammatory, or rejection responses [107]. Additionally, in certain applications like internal adhesion, biodegradability can be advantageous [108,109]. Consequently, polymers such as PVA and carbohydrate polymers are often preferred [110,111]. Although these polymers are commonly used as gel materials, which typically have low toughness, they offer excellent stretchability and adaptability—qualities that are beneficial for use in biological systems [112,113]. Furthermore, since the internal body environment is relatively stable, the issues related to the rapid property changes in gels under varying humidity levels are less significant.

**Figure 10:**
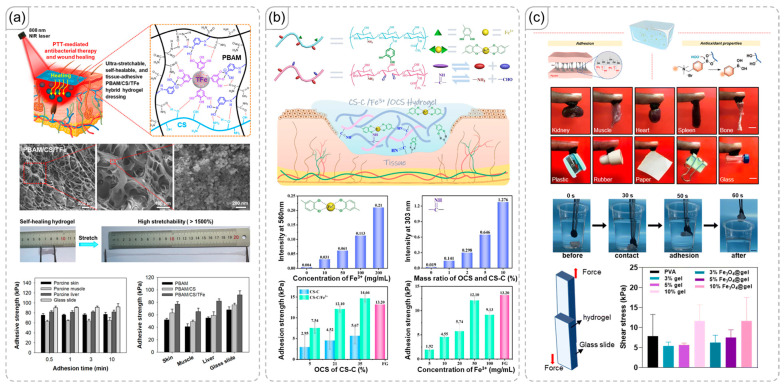
(**a**) Chitosan-based bioadhesive material showing excellent adhesion across multiple substrates, from glass slides to skin, muscle, and liver [114]. Copyright 2022, American Chemical Society. (**b**) Influence of network morphology on the adhesion performance of the material, and comparation in adhesive strength between discussed materials and fibrin glue (pink columns) [115]. Copyright 2025, Elsevier. (**c**) Utilization of boronate ester dynamic covalent bonds for reactive oxygen species (ROS) scavenging in the material [119]. Copyright 2025, Royal Society of Chemistry.

For instance, Yang et al. published research on an adhesive material that demonstrated excellent adhesion to skin and facilitated a wound-healing environment (Figure 10a) [114]. Their work was based on chitosan, a carbohydrate polymer, and a polymer synthesized from acrylamide, both of which possess desirable properties for this application. The network provided an exceptional amount of hydrogen bonding, contributing to adhesion and interaction with fillers. In contrast, Zhao et al. used chitosan as the base material for their study (Figure 10b) [115]. They made two modifications: oxidation to generate aldehyde groups and catechol modification to introduce hydroxyl groups. These modifications facilitated catechol–Fe^3+^ interactions and the formation of amine-based dynamic covalent bonds. The combination of covalent and non-covalent bonds resulted in remarkable adhesive strength, significantly surpassing that of networks containing only one type of interaction. While hydrogen bonding was not emphasized in their manuscript, it likely still exists within the network and contributes to its overall performance.

In bio-adhesive materials, adhesives often serve additional functions, such as providing antioxidant or antibacterial properties, or even enabling magnetism for remote control [116,117,118]. To facilitate these functions, various fillers are commonly incorporated, as previously described. Chitakunye et al. explored boronate ester formation in a novel way (Figure 10c) [119]. The authors noted that the abundance of hydroxyl groups of the network allows it to form boronate esters with N1-(4-boronobenzyl)-N3-(4-boronophenyl)-N1,N1,N3,N3-tetramethylpropane-1,3-diaminium (tsPBA). These bonds contribute to the ability to scavenge reactive oxygen species (ROS) of the gel, as demonstrated using methylene blue. While the adhesion in this study mainly relied on hydrogen bonding, and the dynamic covalent bonds did not directly affect adhesion, the research underscores the potential for further development of dynamic covalent bonds in other applications.

## 5. Conclusions

Dynamic covalent bond-containing adhesive polymers have emerged as powerful tools for advancing adhesive techniques. By integrating dynamic covalent bonds, these materials gain valuable properties such as self-healing, reprocessing, and, crucially, detachment on demand, enhancing their environmental sustainability. Additionally, non-covalent interactions within polymer networks significantly contribute to their exceptional adhesive performance. These interactions not only improve interfacial adhesion, but also enhance the mechanical robustness and energy dissipation capability of the adhesive materials. While the concept of combining dynamic covalent bonds and non-covalent interactions may seem straightforward, ongoing development in this area continues to yield innovative methods for their application. Recent studies have shown that this strategy can provide a useful platform for designing adhesives with both strong bonding performance and adaptive functionalities. Many potential functionalities of these adhesive polymers remain unexplored, and as technology advances, new applications are becoming increasingly appealing for research. However, the current body of literature is still relatively limited, and a more comprehensive understanding of the relationship between network structure, bond dynamics, and adhesive performance is still needed. Ultimately, the synergy between dynamic covalent bonds and non-covalent interactions presents ample opportunities for further development and investigation. Another interesting idea for the development of new adhesive materials is polypeptides, which have been displaying excellent performances as bio adhesive materials [120,121]. Though there are difficulties in materials modifications in order to introduce dynamic covalent bonds into the network and high cost of polypeptides, they still have high potential to be further investigated [122,123].

## Data Availability

No new data were created or analyzed in this study.
